# Thymic Carcinoid in a Patient with Concurrent Manifestations of Multiple Endocrine Neoplasia Type 1

**DOI:** 10.1155/2023/8801080

**Published:** 2023-12-11

**Authors:** Jasmine Zhu, Samantha Dean, Umbreen Hafeez, Sandra Neoh

**Affiliations:** ^1^Department of Endocrinology, Austin Health, 145 Studley Rd, Heidelberg, VIC 3084, Australia; ^2^Melbourne Medical School, University of Melbourne, Parkville, VIC 3010, Australia; ^3^Department of Oncology, Austin Health, 145 Studley Rd, Heidelberg, VIC 3084, Australia; ^4^School of Cancer Medicine, La Trobe University, Plenty Road and Kingsbury Dr, Bundoora, VIC 3086, Australia

## Abstract

Thymic carcinoid tumours, especially in the context of multiple endocrine neoplasia type 1 (MEN 1), present significant clinical challenges due to their rarity and aggressive nature. This case report describes a complex patient with MEN 1, who suffered from multiple manifestations of the disease, including thymic carcinoid. The tumour was initially resected and treated with adjuvant radiotherapy. Due to slow progression over the years, the tumour was treated with two lines of chemotherapy before the patient succumbed to progressive disease. There is currently limited evidence favoring any specific medical treatment for thymic carcinoid.

## 1. Introduction

Thymic carcinoid is difficult to treat given the limited evidence base guiding therapeutic options [1]. When it occurs in the context of MEN 1, it follows a particularly aggressive course [2–4]. We present a complex case of thymic carcinoid occurring in the context of many concurrent manifestations of MEN 1.

## 2. Case Presentation

A 54-year-old man of Chinese descent presented in 2008 with a mediastinal mass due to an atypical carcinoid tumour. He had a history of primary hyperparathyroidism and hypertension. He underwent a radical resection followed by adjuvant radiotherapy. Histopathology demonstrated an atypical thymic carcinoid (mitosis 7/10 HPF) comprising of organoid nests and trabeculae of uniform, small- to medium-sized cells with round to oval nuclei, invading thymic parenchyma but without definite invasion of perithymic fat. Vascular invasion was present, and tumour was present at resection margins (TXN0M0). Genetic testing revealed an *MEN1* gene mutation c.1378C > *T* (p.Arg460X). His daughter, paternal uncle, and paternal aunt were also positive for the mutation, while his son was negative for the mutation.

In 2010, the patient underwent debulking for a presumed large mediastinal recurrence but was instead found to have an abscess, which was treated with antibiotics. The residual tumour continued to slowly progress. The case was discussed at several multidisciplinary meetings. Due to previous extensive surgery and radiation, the recurrence was not amenable to further curative surgery. Overlap in the radiation fields precluded delivery of definitive radiation doses, and the short disease-free interval following prior radiotherapy implied radiation-refractory disease. Peptide receptor radionuclide therapy (PRRT) was deemed unsuitable as there was low somatostatin receptor uptake (Krenning score 1) in the mediastinum on Gallium-68 DOTATATE positron emission tomography (GATATE-PET) imaging performed in 2017. The management at this time was surveillance, given the low volume of asymptomatic disease and restrictions on reimbursement for somatostatin analogues in Australia.

In February 2019, on a routine follow-up fluorodeoxyglucose positron emission tomography (FDG-PET) scan, the patient was found to have significant disease progression in the mediastinum, neck, supraclavicular fossa, bilateral pulmonary hila, pleura, and left perinephric area. The extent of FDG uptake indicated a poorly differentiated neuroendocrine tumour, and the patient commenced first-line palliative chemotherapy with capecitabine and temozolomide in November 2019. He had good disease control for eight months until he experienced significant disease progression in the mediastinal mass in July 2020. Over the past few years, chromogranin A levels progressively increased from 266 *µ*g/L to 603 *µ*g/L (27–94).

The patient also exhibited other manifestations of multiple endocrine neoplasia type 1 (MEN 1). He had severe primary hyperparathyroidism complicated by renal calculi and osteopenia, and a parathyroidectomy was performed overseas in 2002. Due to his previous surgery and radiotherapy, he was unable to undergo a subtotal or total parathyroidectomy. Therefore, a minimally invasive parathyroidectomy was performed for a right-sided parathyroid adenoma in 2018. The patient had a preoperative corrected calcium level of 2.82 mmol/L (2.10–2.60) accompanied by a parathyroid hormone level of 22.4 pmol/L (1.6–6.0). A biochemical resolution was not achieved postoperatively, with a day 6 corrected calcium level of 2.89 mmol/L and parathyroid hormone level of 20.3 pmol/L. His ongoing primary hyperparathyroidism continued to be observed without any specific treatment.

Magnetic resonance imaging (MRI) performed in 2017 detected a 3 mm pituitary lesion, but the imaging was unable to be repeated due to severe claustrophobia. The patient had acromegalic facial features and enlarged hands. Pituitary function testing during his admission showed a raised growth hormone (GH) level of 11.45 *µ*g/L (<0.97), insulin-like growth factor 1 of 44.01 nmol/L (8.06–27.82), and prolactin of 473 mIU/L (56–278) (Table 1), raising the possibility of a growth hormone-secreting adenoma. Repeat testing yielded similar results, and retrospective review of the medical records revealed that he had mildly elevated prolactin, GH, and IGF-1 levels since 2008.

In 2008, the patient had a distal pancreatectomy and splenectomy performed for a 60 mm nonfunctioning pancreatic neuroendocrine tumour at an external institution. Histology demonstrated a pancreatic islet cell tumour with a low mitotic rate (2–10/10 HPF), no necrosis, and tumour present at the resection margins. Immunostaining showed tumour cells positive for synaptophysin and cytokeratin (AE1/AE3), and Ki-67 showed a very low labelling index (less than 2% Ki-67+ cells). Therefore, the tumour was classified as a well-differentiated endocrine neoplasm of uncertain behaviour. Staining was negative for insulin and somatostatin, with a small subpopulation strongly positive for glucagon. The patient had a normal serum glucagon level of 123 pg/mL (40–140) at the time. Serial imaging revealed new DOTATATE avid lesions in the pancreas in 2016 and in the liver in 2017 and 2019. Additionally, the patient presented with hypovolemic shock secondary to a bleeding duodenal ulcer in 2010, accompanied by mildly elevated gastrin levels of 189–284 pmol/L (6–55), raising the possibility of a gastrinoma.

In July 2020, the patient presented to the emergency department with dysphagia secondary to the local progression of the mediastinal tumour requiring gastroscopy removal. A computer tomography (CT) scan showed that the mediastinal mass was compressing the trachea and oesophagus and obstructing the superior vena cava (SVC), with a new thrombus in the pulmonary trunk. He was treated with dexamethasone and anticoagulated with enoxaparin. He also received palliative radiotherapy to the mediastinum.

Despite receiving high doses of dexamethasone, he was noted to be hypoglycaemic, with a fasting plasma glucose level of 2.1 mmol/L (3.9–5.6), C-peptide of 0.99 nmol/L (0.33–1.47), insulin of 4.0 mU/L (1.9–23.0), and proinsulin of 37.5 pmol/L (<13.3), raising the suspicion of a proinsulin-secreting insulinoma. He was minimally symptomatic. This was treated with oral cornstarch, continuous intravenous 5% dextrose, boluses of 50% dextrose, and diazoxide 50 mg TDS, which partially attenuated the hypoglycaemia.

The patient developed worsening peripheral edema secondary to the SVC obstruction, dexamethasone, and diazoxide. Due to deteriorating performance status, in August 2020, he was commenced on reduced dose palliative carboplatin and etoposide chemotherapy. He was also switched from diazoxide to octreotide 100 mcg BID to minimise his edema.

After his condition stabilised, he was discharged home and switched to long-acting octreotide. He tolerated the second cycle of chemotherapy poorly, requiring readmission with lower limb cellulitis, pancytopenia, and nocturnal hypoglycaemia. Due to further deterioration with pain and facial edema, he was transferred to the palliative care unit and commenced on a syringe driver with hydromorphone and midazolam, but subsequently made a remarkable recovery, and was discharged home.

**Figure 1:**
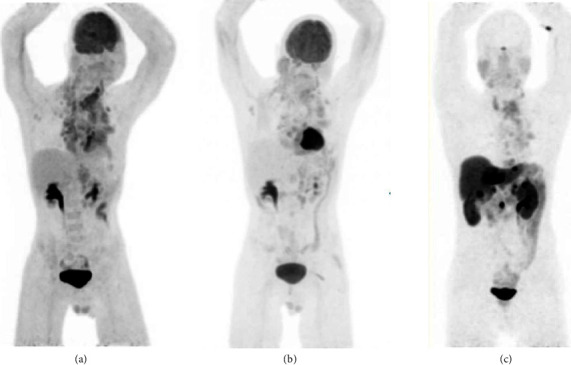
Fluorodeoxyglucose (FDG). (a) August 2020 and (b) December 2020 versus Gallium-68 DOTATATE (GATATE), and (c) positron emission tomography (PET). FDG avidity of the mediastinal mass and left perinephric mass implies a more aggressive and less differentiated disease. DOTATATE avidity of liver and pancreatic lesions implies well-differentiated neuroendocrine tumours.

**Figure 2:**
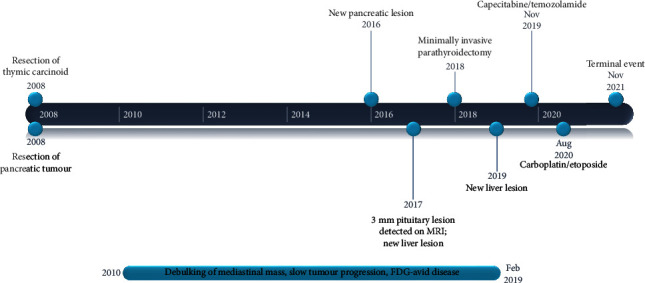
The course of disease.

The patient only completed two cycles of carboplatin and etoposide due to various adverse effects of chemotherapy. However, he demonstrated a marked clinical response, with resolution of the SVC obstruction and facial edema, and reduced FDG uptake in the mediastinum and neck (Figure 1). An FDG-PET demonstrated ongoing stable disease one year later. Several months later, the patient again had increasing presentations with impacted food boluses requiring gastroscopy removal, with a CT chest demonstrating increased fibrosis. His case was discussed in a multidisciplinary meeting and thought to be unsuitable for an oesophageal stent. In November 2021, the patient was brought to the emergency department via ambulance with end-stage dyspnea and decreased conscious state; comfort measures were implemented and he died. He was subsequently found to have a pneumothorax. The sequence of events is depicted in Figure 2.

## 3. Discussion

Thymic carcinoids are rare neoplasms of the thymus with neuroendocrine differentiation, typically presenting as a mass in the anterior mediastinum. One-third to half of cases are secretory [5], with ACTH being the most common hormone secreted. These tumours have also been reported to secrete growth hormone-releasing hormones [6]. Carcinoid syndrome is very rare. Thymic carcinoids of low grade are classified as typical carcinoids, and those of intermediate grade are classified as atypical carcinoids [7].

MEN 1 is a rare autosomal dominant condition that predisposes patients to multiple tumours of the endocrine glands [8]. Thymic carcinoids occur in 2-3% of patients with MEN 1 [2, 3], and a quarter of all thymic carcinoids occur due to MEN 1 [9]. Thymic carcinoids are typically considered a late manifestation of MEN 1, with the median and mean age of diagnosis in previous literature ranging from 40 to 44 years [2, 9]. Studies have found that they occur predominantly in males [3, 9] and are strongly associated with smoking [4, 9]. Compared with those that occur sporadically, thymic carcinoids occurring in the context of MEN 1 follow a particularly aggressive course and poor prognosis, with 10-year survival ranging from 25 to 45% [2–4].

The pathophysiology of thymic carcinoids is unclear. Unlike many other MEN 1-related tumours, thymic carcinoids do not demonstrate any loss of heterozygosity at the *MEN1* gene locus [9]. However, one series [9] found that two out of seven tumours had a loss of heterozygosity at chromosome 1p, which contains genes essential in the development of other tumours, and it has been hypothesised that perhaps there are modifier genes that interact with other factors to precipitate the development of thymic carcinoid.

It is common practice at our institution to perform a prophylactic thymectomy at the same time as performing a parathyroidectomy in patients with MEN 1. This is usually done through a cervical collar incision, which only removes the superior aspect of the thymus. A transthoracic approach would be able to access the mediastinal part of the thymus but has a greater risk of surgical complications. There are no randomised data to support this practice, and in fact, there have been reports of patients who developed thymic carcinoid after having a prophylactic thymectomy through a transcervical approach [2]. However, a retrospective study found a significant association between having a prophylactic transcervical thymectomy and prevention of the development of thymic carcinoid [3]. Another advantage of this procedure is that there are often supernumerary or ectopic parathyroid glands in the thymus, which can be removed at the same time. Therefore, despite the limitations of available data, this procedure continues to be routinely performed at our institution.

Surgical resection should be considered for localised disease, and radiotherapy can improve control [1, 10]. In terms of disseminated thymic neuroendocrine tumours, there have been small retrospective series demonstrating some response to temozolomide [11], platinum-based chemotherapy [11], and everolimus [12]. More investigational therapeutic options include somatostatin analogues, peptide receptor radionuclide therapy, and pasireotide, and these have been summarised in a review by Lang et al. [12]. It is important to note that there is limited evidence available to favour one treatment regimen over another [1].

## 4. Conclusions

This case has highlighted not only the difficulties of treating thymic carcinoid but also the complexities of managing a patient with multiple concurrent endocrine issues. Despite the very aggressive nature of thymic carcinoids, survival is possible for many years after the initial diagnosis.

## Figures and Tables

**Table 1:** Endocrine panel measurements.

Parameter	Measurement	Units	Reference range
Prolactin	473	mIU/L	56–278
Growth hormone	11.45	*µ*g/L	<0.97
Insulin-like growth factor 1	44.01	nmol/L	8.06–27.82
Thyroid-stimulating hormone	1.25	mU/L	0.38–5.30
Thyroxine	10.20	pmol/L	7.9–14.4
Luteinising hormone	8.4	IU/L	1.3–8.6
Testosterone	11.5	nmol/L	9–28.3
Cortisol	228	nmol/L	185–624
Adrenocorticotropic hormone	12.1	ng/L	7.2–63.3
Serum corrected calcium	2.89	mmol/L	2.10–2.60
Parathyroid hormone	20.3	pmol/L	1.6–6.0
Glucose	2.1	mmol/L	3.9–5.6
C-peptide	0.99	nmol/L	0.33–1.47
Insulin	4.0	mU/L	1.9–23.0
Proinsulin	37.5	pmol/L	<13.3
Gastrin	189–284	pmol/L	6–55
Chromogranin A	266–603	*µ*g/L	27–94

## Data Availability

The data used to support the findings of this study are included within the article.
